# A Retrospective Assessment of Computed Tomography-Based Body Composition and Toxicity in Ovarian Cancer Patients Treated with PARP Inhibitors

**DOI:** 10.3390/cancers17121963

**Published:** 2025-06-12

**Authors:** Marta Nerone, Giorgio Raia, Maria Del Grande, Lucia Manganaro, Giordano Moscatelli, Clelia Di Serio, Andrea Papadia, Esteban Ciliberti, Elena Trevisi, Cristiana Sessa, Filippo Del Grande, Ilaria Colombo, Stefania Rizzo

**Affiliations:** 1 Istituto Oncologico della Svizzera Italiana (IOSI), Ente Ospedaliero Cantonale (EOC), Via A. Gallino, 6500 Bellinzona, Switzerlandilaria.colombo@eoc.ch (I.C.); 2 Istituto di Imaging della Svizzera Italiana (IIMSI), Ente Ospedaliero Cantonale (EOC), Via Tesserete 46, 6900 Lugano, Switzerlandstefaniamariarita.rizzo@eoc.ch (S.R.); 3 Department of Rheumatology, University Hospital Basel, 4031 Basel, Switzerland; 4 Department of Radiological, Oncological and Pathological Sciences; University of Rome Sapienza, 00185 Roma, Italy; 5 CTU (Clinical Trial Unit), Ente Ospedaliero Cantonale, 6900 Lugano, Switzerland; 6 Facoltà di Scienze biomediche, Università della Svizzera italiana, Via Buffi 13, 6900 Lugano, Switzerland; 7 Department of Gynecology and Obstetrics, Ente Ospedaliero Cantonale (EOC), Via Tesserete 46, 6900 Lugano, Switzerland

**Keywords:** body composition, computed tomography, ovarian cancer, toxicity, PARP inhibitors

## Abstract

**Objectives:** The objective of this single-site retrospective study was to assess the association between Computed Tomography (CT)-based whole-body composition values with dose reduction in patients with a diagnosis of epithelial ovarian cancer (EOC) treated with poly ADP-ribose polymerase (PARP) inhibitors (PARPi). **Methods**: Forty-eight patients (median age 61 years; interquartile range 53–68.5) with EOC who had a thorax and abdomen CT scan (performed before starting PARPi) were enrolled. Recorded clinical data included age, weight, height, stage, start and end date of PARPi, dose reduction, premature discontinuation of therapy, date of last contact, progression, and death. Body composition values were automatically extracted by dedicated software. Given the exploratory nature of the study, the statistical analysis combined univariate assessments (univariate logistic regression) used to evaluate the individual effect of each variable on the probability of dose reduction, with a classification tree approach—a data-driven machine learning method considering all variables simultaneously as covariates. This integrated strategy was designed to identify empirical cut-offs defining body composition profiles associated with increased risk of toxicity. **Results**: Univariate logistic regression showed no statistically significant effect of body composition variables on the probability of dose reduction. Due to the complexity of variable relations, a machine-learning approach with a classification tree showed that SKM (skeletal muscle) was the sole body composition variable significantly associated with dose reduction. Specifically, there was a higher risk of dose reduction with SKM values ≥ 7506 cm^3^ and < 8650 cm^3^ (*p* = 0.0118). **Conclusions**: In this exploratory study, a significant association of whole-body composition parameters (SKM) with dose reduction was observed in patients with a 7506 cm^3^ ≤ SKM < 8650 cm^3^. If confirmed in larger cohorts, these findings could help clinicians identify patients who might benefit from an upfront reduced PARPi dose.

## Simple Summary

Poly ADP-ribose polymerase (PARP) inhibitors (PARPi) are a well-established maintenance therapy in stage III and IV ovarian cancer, with evidence of efficacy, especially in patients with germline and/or somatic pathogenic variants (PVs) in the *BRCA1/2* genes and in patients with homologous recombination deficiency. While the toxicity profile of PARPi—often leading to dose reductions—is well characterized in both clinical trials and real-world settings, the potential link between drug toxicity and body composition parameters remains unexplored. This exploratory study aims to investigate that potential association, with the goal of identifying a specific patient profile more susceptible to treatment-related toxicity.

## 1. Introduction

Epithelial ovarian cancer (EOC) is the second most lethal gynecological malignancy in developed countries, with an estimated 19,680 new cases and 12,740 deaths in the United States in 2024 [1]. Despite significant advancements in disease treatment, prognosis remains poor, with a 5-year overall survival of 20% in patients with stage IV disease [2]. The introduction of poly(ADP-ribose) polymerase (PARP) inhibitors (PARPi) into clinical practice has marked a significant advancement in the treatment of EOC, particularly for patients harboring germline and/or somatic pathogenic variants in the *BRCA1* or *BRCA2* genes, or in tumors with homologous recombination deficiency (HRD) [3,4]. Several clinical trials have demonstrated improvements in progression-free survival (PFS) and some in overall survival (OS) with the use of PARPi as maintenance therapy after post-operative chemotherapy or after second-line chemotherapy [3,5,6,7]. Olaparib is currently approved as maintenance therapy for 2 years following first-line platinum-based chemotherapy in patients who respond to chemotherapy and harbor *BRCA1/2* pathogenic variants [3] or in combination with bevacizumab for HRD-positive tumors [4]. Niraparib and rucaparib are approved as maintenance therapy following first-line platinum-based chemotherapy, irrespective of BRCA and HRD status [8,9]. Additionally, before the approval for the first-line maintenance treatment, PARPi were used as maintenance therapy following response to second-line chemotherapy in patients with platinum-sensitive recurrence, regardless of *BRCA1/2* or HRD status [10,11]. It is, therefore, expected that the majority of women diagnosed with EOC, particularly those with high-grade serous carcinoma, will receive a PARPi during their treatment course.

The most common side effects of PARPi include hematological toxicities (especially anemia, with rare cases of myelodysplasia or progression to acute myeloid leukemia), gastrointestinal toxicity (primarily nausea), and fatigue. Prompt recognition and management of PARPi-related toxicities are crucial to ensure patient adherence to treatment, as these therapies are typically administered up to 2 years (in the case of first-line olaparib or rucaparib maintenance), 3 years (for first-line niraparib maintenance), or until progression (for second-line maintenance).

Imaging examinations, including Computed Tomography (CT), are regularly performed at initial preoperative staging and during the follow-up for gynecological cancers, including ovarian cancer patients [12]. Moreover, in patients with EOC who are candidates for a PARPi, a CT scan is required prior to initiating maintenance therapy to confirm that complete remission (CR), partial response (PR), or non-evidence of disease (NED) is achieved at the end of chemotherapy, as per the indication for starting a PARPi. CT is also considered a reference method to assess muscle quantity and quality, as well as adipose tissue distribution, in a non-invasive way [13]. Its use is increasingly advocated as an opportunistic screening to evaluate body composition. Currently, several software packages allow the extraction of quantitative features from a single axial CT image, usually at the level of the third lumbar vertebra (L3), including skeletal muscle area (SMA), subcutaneous adipose tissue (SAT), skeletal muscle density (SMD) and visceral adipose tissue (VAT). From these measurements, the body composition parameters are frequently approximated for the whole body, dividing the single axial value by the height square. Furthermore, advancements in segmentation software and extraction of quantitative features may offer the possibility of a complete automatic volumetric quantification of body composition profiling from the entire CT scan, thus offering a direct and complete body composition evaluation without the need for approximate estimates [14]. Previous studies have evaluated the association of body composition and survival in ovarian cancer patients [14,15,16] with conflicting results. Furthermore, only a few studies have explored the association between body composition and chemotoxicity in ovarian cancer patients [17,18,19], and none have focused specifically on patients treated with PARPi. The primary objective of this exploratory study was to assess whether volumetric automatic quantification of body composition values extracted from routinely performed CT scans is associated with toxicity in ovarian cancer patients treated with PARPi.

## 2. Materials and Methods

### 2.1. Patients Selection

From a database of patients with a diagnosis of EOC referred to our institution, we selected patients who received a PARPi between February 2017 and July 2023, and had a pretreatment CT scan (PET with CT contrast enhancement was permitted), including both thorax and abdomen in one acquisition after contrast injection. The main inclusion criteria were age ≥ 18 years, diagnosis of EOC, treatment with a PARPi as maintenance at first diagnosis or at recurrence, and a CT scan performed before starting PARPi. The main exclusion criteria were any previous or concurrent malignancy (except concurrent diagnosis of breast cancer in *BRCA1/2* mutant patients) and the presence of technical problems on the CT images, such as metallic prostheses [20].

### 2.2. Clinical Data Recorded

We recorded age at diagnosis; weight and height to calculate body mass index (BMI); International Federation of Gynecology and Obstetrics (FIGO) stage; start and end date of PARPi therapy; date of recurrence, if any; dose reduction, recorded as reduction compared to initial dose; premature discontinuation of therapy due to toxicity; treatment interruption due to therapy-induced adverse events. We also collected the date of last contact and/or date of progressive disease at CT scan or PET/CT.

### 2.3. Extraction of Volumetric Body Composition Features

CT series used for volumetric automatic segmentation were acquired after contrast medium in the portal venous phase and analyzed through a dedicated software (DAFS, version 3.0 Voronoi Health Analytics Inc, Canada) that automatically segments each scan in folders and provides a report of the values selected for segmentation. In this study, we specifically expressed the following tissues and organs as volumes (cm^3^): skeletal muscle (SKM); intramuscular adipose tissue (IMAT); visceral adipose tissue (VAT); subcutaneous adipose tissue (SAT); visceral and subcutaneous adipose tissue (VAT-U-SAT); epicardial adipose tissue (EpAT); paracardial adipose tissue (PaAT); thoracic adipose tissue (ThAT); bone; liver (LIV); spleen (SPL); aortic calcification (AOC); heart (HRT). The segmentations were visualized and checked in the three planes.

### 2.4. Statistical Analysis

For continuous variables, we reported median and interquartile range (IQR). The distribution of each body composition variable was assessed to evaluate normality and asymmetry using the Kolmogorov–Smirnov test, skewness coefficients, and the median absolute deviation (MAD), given the presence of outliers.

Univariate logistic regression was performed to estimate odds ratios and assess the association between each variable and the probability of dose reduction. In parallel, multicollinearity among covariates was evaluated using appropriate indicators such as Variance Inflation Factor (VIF) and correlation matrices.

The combined assessment of distributional characteristics, univariate associations, and multicollinearity guided the decision on whether a multivariate logistic regression model was methodologically justified and statistically meaningful.

To explore complex and potentially non-linear relationships among variables, a classification tree model with a binary outcome (dose reduction: yes/no) was also implemented. This machine learning approach allowed for simultaneous consideration of all covariates, identification of data-driven cut-offs, and classification of patients according to body composition profiles associated with increased risk of dose reduction and recurrence [21].

Finally, General Linear Models (GLMs) were applied to estimate the association between dose reduction and the three skeletal muscle (SKM) categories identified through the classification tree analysis.

All calculations and statistical analysis were performed with R 4.3.0 (R Core Team, 2023) [22].

## 3. Results

A total of 48 patients met the inclusion and exclusion criteria (Table 1). Median age at diagnosis was 61 (IQR 53; 68.5); most patients had a diagnosis of high-grade serous ovarian carcinoma (*n* = 43; 90%), and 35 patients underwent surgery at diagnosis (73%). Most patients were diagnosed with stage IIIC (*n* = 20; 42%) and IV (*n* = 19; 40%). All patients had received platinum-based chemotherapy prior to maintenance therapy with a PARPi in accordance with current standards of care. All patients performed a CT scan prior to the initiation of PARPi (mean 25.68 days, with a standard deviation of 20.9). Of the 48 total patients, 16 (33%) received a PARPi in the first-line setting, and 32 (67%) received it in subsequent lines. No patients received the PARPi as a single-line monotherapy. The majority of patients received olaparib (*n* = 38; 79%). For the 10 patients who received niraparib, the prescribing recommendations based on weight and platelet count were followed. Specifically, in patients with body weight ≥ 77 kg and with a baseline platelet count ≥ 150,000/μL, the recommended initial dose is 300 mg, and in our cohort, one patient received 300 mg as a niraparib daily dose. The median duration of treatment with a PARPi was 17.1 months (IQR 5.0; 22.5 months). Median follow-up was 25.6 months (IQR 11.7;34.7).

Overall, 20 patients (42%) receiving a PARPi experienced a dose reduction due to drug-related toxicity. Of the 20 patients who required a dose reduction, 10 (50%) experienced toxicity leading to dose modification within the first six months of treatment. We did not observe meaningful differences in dose reduction rates across the different lines of maintenance PARPi therapy. The most common toxicity observed was hematologic, including thrombocytopenia and anemia, followed by gastrointestinal toxicity (nausea, vomiting) and fatigue. Additionally, seven patients (15%) had a permanent treatment discontinuation due to toxicity.

The descriptive plots of body composition variables stratified according to the presence and absence of dose reduction are shown in Table 2.

**Figure 1:**
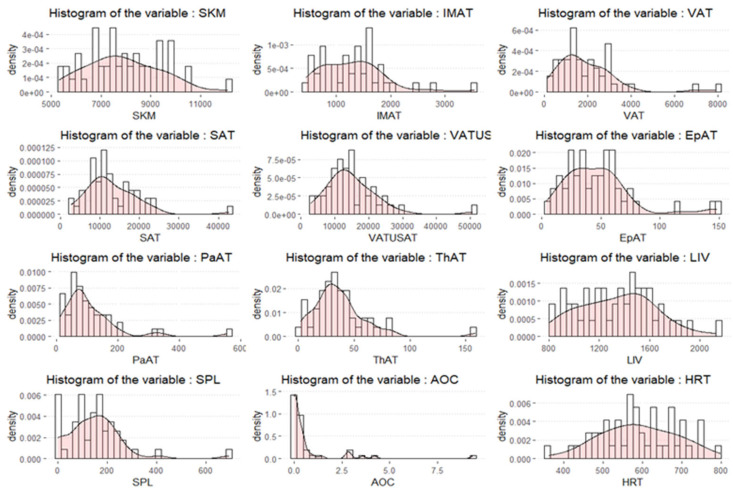
Histogram plots for the distribution of body composition variables.

The histogram plots showed that the body composition volumetric variables may be asymmetrically distributed (Figure 1).

The univariate logistic regression analyses assessing the association between body composition variables and the occurrence of dose reduction did not reveal any statistically significant effects (Table 3). Given the absence of significant associations and the presence of substantial multicollinearity among the variables, a multivariate logistic regression model was not performed, as it would not have provided additional interpretability or robustness in this exploratory context.

The classification tree, including age and BMI, showed that the SKM was the sole variable significantly associated with dose reduction. Specifically, three categories were identified based on two SKM values (7506 cm^3^ and 8650 cm^3^), as follows: group one: SKM≥ 8650 cm^3^; group two: 7506 ≤ SKM < 8650 cm^3^; group three: SKM < 7506 cm^3^. Among these groups, the highest risk for dose reduction was seen in group two, where it was more than twice the risk of the reference category (group 1) (*p* = 0.0118) (Table 4).

## 4. Discussion

In this study, we evaluated the correlation between body composition parameters and PARPi toxicity in patients with EOC receiving maintenance. We demonstrated that SKM was the only body-composition variable significantly associated with PARPi dose reduction. Specifically, three categories were defined based on two SKM threshold values (7506 and 8650 cm^3^) and according to a machine learning approach: group one (SKM ≥ 8650 cm^3^), group two (7506 ≤ SKM < 8650 cm^3^), and group three (SKM < 7506 cm^3^). Comparing these groups, the highest risk of dose reduction was observed in group two, where the risk was more than twice of the reference category (group one), with a *p*-value of 0.0118.

Since toxicity can occur through multiple adverse events and varying grades of severity, dose reduction was considered a surrogate marker for clinically significant toxicity.

Patients with high SKM did not have an increased risk of PARPi dose reduction, possibly due to both metabolic and pharmacokinetic factors. One hypothesis is that PARP1, the enzyme targeted by PARPi, is highly expressed in muscle tissue [23,24,25]. Greater SKM could mean a larger muscle tissue volume where PARPi can accumulate and be metabolized. As a result, in patients with higher muscle mass, the bioavailability of PARPi in the bloodstream is lower, reducing the likelihood of drug toxicity.

In contrast, patients with low SKM may experience a higher proportion of free-circulating drugs. Although we did not observe hypoalbuminemia (which could indicate sarcopenia), lower SKM might correlate with lower plasma albumin levels. Since olaparib has a 56% plasma protein binding rate, decreased albumin levels could result in a higher fraction of plasma-free olaparib [26]. This free drug fraction is more readily eliminated by kidneys, thus potentially reducing bioavailability and the likelihood of toxicity [27].

For patients with intermediate SKM values, the effect of reduced muscle accumulation of the PARPi, along with its distribution volume, may result in moderate exposure to the drug. These patients may not benefit from either the protective effect of a high SKM or the potential protection of lower plasma protein binding, which can be assumed in patients with lower SKM. This could potentially explain why these patients had a higher likelihood of requiring dose reduction due to toxicity.

Previous studies have shown that reduced the skeletal muscle area index (SMI) is associated with poorer progression-free survival (PFS) in ovarian cancer patients treated with PARPi, and a higher SMI correlates with a lower risk of disease progression [28]. No prior analyses have examined how SMI may correlate to PARPi pharmacodynamics or toxicity-related dose reduction. Thus, further research is needed to explore how skeletal muscle mass influences the pharmacokinetics and toxicity profiles of PARPi, offering potential insights for personalized treatment strategies.

In our experience, the dose reduction rate in patients receiving PARPi therapy was 41%. This is consistent with data from both clinical trials [3,7,8,9,11,29,30,31,32] and real-world studies [33,34,35,36,37].

This study includes a cohort of 48 patients, providing a valuable basis for an exploratory analysis of the relationship between body composition parameters and PARPi toxicity. While the sample size and cohort heterogeneity do not allow for definitive conclusions, the findings should be viewed as hypothesis-generating, offering initial insights into potentially meaningful clinical associations.

Our results suggest that patients with higher skeletal muscle mass (SKM) may be less prone to toxicity and dose reductions. This observation could support the notion that, in clinical practice, full-dose PARPi treatment might be more safely maintained in these patients.

Although body composition analysis tools are not yet routinely implemented in clinical settings, their use is expected to grow—especially through opportunistic assessments in patients undergoing CT scans for standard care. Such integration could provide valuable information to support personalized treatment planning and monitoring based on individual body composition profiles.

## 5. Conclusions

The data from our study suggest that patients with epithelial ovarian cancer receiving PARPi are more likely to experience dose reductions due to drug toxicity when presenting with median SKM levels. Given the limited sample size in this study, it is essential to validate these hypothesis-generating findings in larger cohorts and in a more homogeneous population (e.g., only first-line maintenance).

Knowledge of body composition parameters could help identify patients at greater risk of PARPi-related toxicities, enabling personalized treatment monitoring and timely dose adjustments.

## Figures and Tables

**Table 1:** Patient characteristics (*n* = 48).

	*N* (%)
Age at diagnosis, median (IQR)	61 (53;68.5)
Histology	
High grade serous	43 (90)
Endometrioid	3 (6)
Clear Cell	1 (2)
Undifferentiated	1 (2)
Surgery	
Primary surgery	35 (73)
Interval debulking surgery	10 (21)
No surgery	3 (6)
FIGO Stage at diagnosis	
IC	2 (4)
IIA	1 (2)
IIIA	2 (4)
IIIB	4 (8)
IIIC	20 (42)
IV	19 (40)
g/s BRCA1/2 status	
g/s BRCA1/2 PV	15 (31)
g/s BRCA1/2 wild type	27 (56)
Untested	6 (13)
PARP inhibitor	
Olaparib	38 (79)
Niraparib	10 (21)
PARP inhibitor setting	
1st line maintenance	16 (33)
2nd line maintenance	24 (50)
3rd line maintenance	6 (13)
4th line maintenance	2 (4)
Dose reduction	
No	28 (58)
Yes	20 (42)
Reasons for dose reduction	
Trombocytopenia (G1-G2)	5
Trombocytopenia G3	1
Anemia (G1-G2)	4
Anemia G3	1
Nausea (G1-G2)	4
Fatigue	4
Disgeusia	1
Increased AST/ALT	1
Vomiting	1
Cough	1
Heart Failure	1
Renal impairment	1
Permanent discontinuation	7 (15)
Anemia G2	3
Trombocytopenia (G1-G2)	1
Fatigue G2	2
Nausea G1	1
Recurence/progression	
No	22 (46)
Yes	26 (54)
BMI, median (IQR)	24 (21.5;28.7)

g: germline, s: somatic, PV: pathogenic variant, BMI: body mass index, G: grade.

**Table 2:** Mean and standard deviation of volumetric body composition variables among patients without and with dose reduction.

	No Dose Reduction	Dose Reduction
*N*	28	20
SKM (mean (SD))	8049.59 (1681.14)	7639.54 (1205.84)
IMAT (mean (SD))	1304.75 (579.91)	1310.40 (672.98)
VAT (mean (SD))	1985.72 (1444.50)	1953.22 (1568.51)
SAT (mean (SD))	12,372.74 (5924.65)	13,888.81 (8048.12)
VAT-U-SAT (mean (SD))	14,358.46 (7037.06)	15,842.03 (9489.89)
EpAT (mean (SD))	48.32 (30.32)	45.75 (29.69)
PaAT (mean (SD))	108.73 (82.84)	108.14 (112.70)
ThAT (mean (SD))	36.61 (21.44)	38.06 (32.09)
LIV (mean (SD))	1371.04 (266.89)	1308.54 (345.58)
SPL (mean (SD))	142.86 (95.24)	164.66 (150.20)
AOC (mean (SD))	0.45 (0.87)	1.13 (2.26)
HRT (mean (SD))	597.89 (102.00)	580.79 (89.68)
AgeD (mean (SD))	60.11 (10.88)	60.80 (11.66)
BMI (mean (SD))	25.18 (5.42)	25.51 (7.92)

All quantitative variables are volumes (cm^3^).

**Table 3:** Univariate logistic regression analysis evaluating impact of body composition volumetric variables on probability of dose reduction.

Variable	Crude OR	(95%CI)	*p*-Value
SKM	0.9998	(0.9994,1.0002)	0.3498
IMAT	1	(0.9991,1.001)	0.9746
VAT	1	(0.9996,1.0004)	0.9396
SAT	1	(0.9999,1.0001)	0.4528
VAT-U-SAT	1	(1,1.0001)	0.5319
EpAT	0.997	(0.9774,1.017)	0.7658
PaAT	0.9999	(0.9939,1.006)	0.983
ThAT	1.0022	(0.9803,1.0245)	0.8478
LIV	0.9993	(0.9973,1.0012)	0.4745
SPL	1.0015	(0.9967,1.0064)	0.5369
AOC	1.3652	(0.8476,2.1989)	0.2005
HRT	0.9981	(0.9921,1.0042)	0.5418

**Table 4:** General linear model estimates of the risk of dose reduction in the three categories previously identified by the classification tree.

	Estimate	Std. Error	*z* Value	Pr (>|*z*|)
SKM ≥ 8650 cm^3^	−1.2993	0.6513	−1.995	0.0461
7506 ≤ SKM < 8650 cm^3^	2.2156	0.8799	2518	0.0118
SKM < 7506 cm^3^	0.6802	0.8025	0.8025	0.3966

## Data Availability

The data presented in this study are available on request from the corresponding author due to privacy restriction.
